# The patterns of response of 11 regimens for infantile spasms

**DOI:** 10.1038/s41598-020-68403-6

**Published:** 2020-07-13

**Authors:** Leilei Mao, Miriam Kessi, Pan Peng, Fang He, Ciliu Zhang, Lifen Yang, Liwen Wu, Fei Yin, Jing Peng

**Affiliations:** 1 Department of Pediatrics, Xiangya Hospital, Central South University, Changsha, China; 2 Hunan Intellectual and Developmental Disabilities Research Center, Changsha, China

**Keywords:** Health care, Neurology

## Abstract

Infantile spasms (ISs) is a devastating form of an early infantile epileptic encephalopathy. The patterns of response of multiple regimens, and the difference in response rates for the cases who receive first-line therapies on time versus those who receive them after non-first-line therapies are unknown. We performed a study involving 314 ISs cases aiming to investigate the patterns of response of 11 regimens, and the difference in response rates for the cases received first-line therapies as first two regimens versus those who received other drugs prior to first-line options. As a result, the efficacy of each regimen was: the foremost two regimens; 36.99%, third; 10.27%, fourth; 6.16%, fifth; 5.48%, and from the sixth regimen onwards, each additional regimen added ≤ 2% probability of seizure freedom. There was a statistically significant difference in seizure freedom rates between cases received first-line therapies as first or second regimen versus those who received them later. Our study revealed for the first time that in ISs cases, seizure freedom is likely to be observed within the first five regimens, and an early administration of first-line therapies is superior to non-first-line options. These results will aid in management of ISs cases.

## Introduction

Infantile spasms (ISs) is an epileptic syndrome which occurs in children younger than 1 year and rarely older than 2 years characterized by clinical spasms, hypsarrhythmia in an electroencephalogram (EEG), and most of the time spasms are accompanied by developmental delay or regression^1^. ISs belongs to the group of drug resistant epilepsy, and has an incidence rate of 0.25–0.42 per 1,000 children^2,3^.

The adrenocorticotropic hormone (ACTH), vigabatrin (VGB) and corticosteroids are the recommended first-line therapies, however, they seem to be effective for some cases^4–6^ as 33–56% of the patients remain with uncontrolled spasms^7,8^. Several antiepileptic drugs (AEDs) have been introduced for the past two decades^9^. Some of those newly introduced AEDs can ameliorate ISs. A recent systematic review revealed that levetiracetam (LEV), topiramate (TPM), zonisamide, sodium valproate (VPA) and benzodiazepines (clonazepam or nitrazepam) might be useful in controlling spasms^10^. The ketogenic diet, modified Atkins diet and surgery have been reported to be effective too^4,10,11^. There is an existence of inconsistent and conflicting preferred treatment options worldwide^10^. Factors such as availability of the recommended drugs, affordability, and adverse effects may explain treatment variation globally^12^.

One study on general epilepsy revealed that 57.3% of the cases achieved seizure freedom with the correct choice of first two AEDs, and the added 3–10 AEDs provided less than 10% increment of seizure freedom^13^. Although first-line therapies for ISs have been clarified, it is not clear whether third, fourth, and an increasing number of regimens can rise the percentage of seizure freedom for non-responders. First-line therapies are not available or affordable in some countries. Consequently, the difference in response rates between cases who receive first-line therapies as the foremost two regimens versus those who receive them after non first-line drugs is not clear. Therefore, we aimed to investigate the patterns of response of 11 regimens, and the difference in response rates for the cases received first-line therapies as first two regimens versus those who received other drugs prior to first-line therapies. Moreover, the prognostic factors for 1-year seizure-freedom, and the determinants of good psychomotor development will be discussed.

## Results

**Figure 1:**
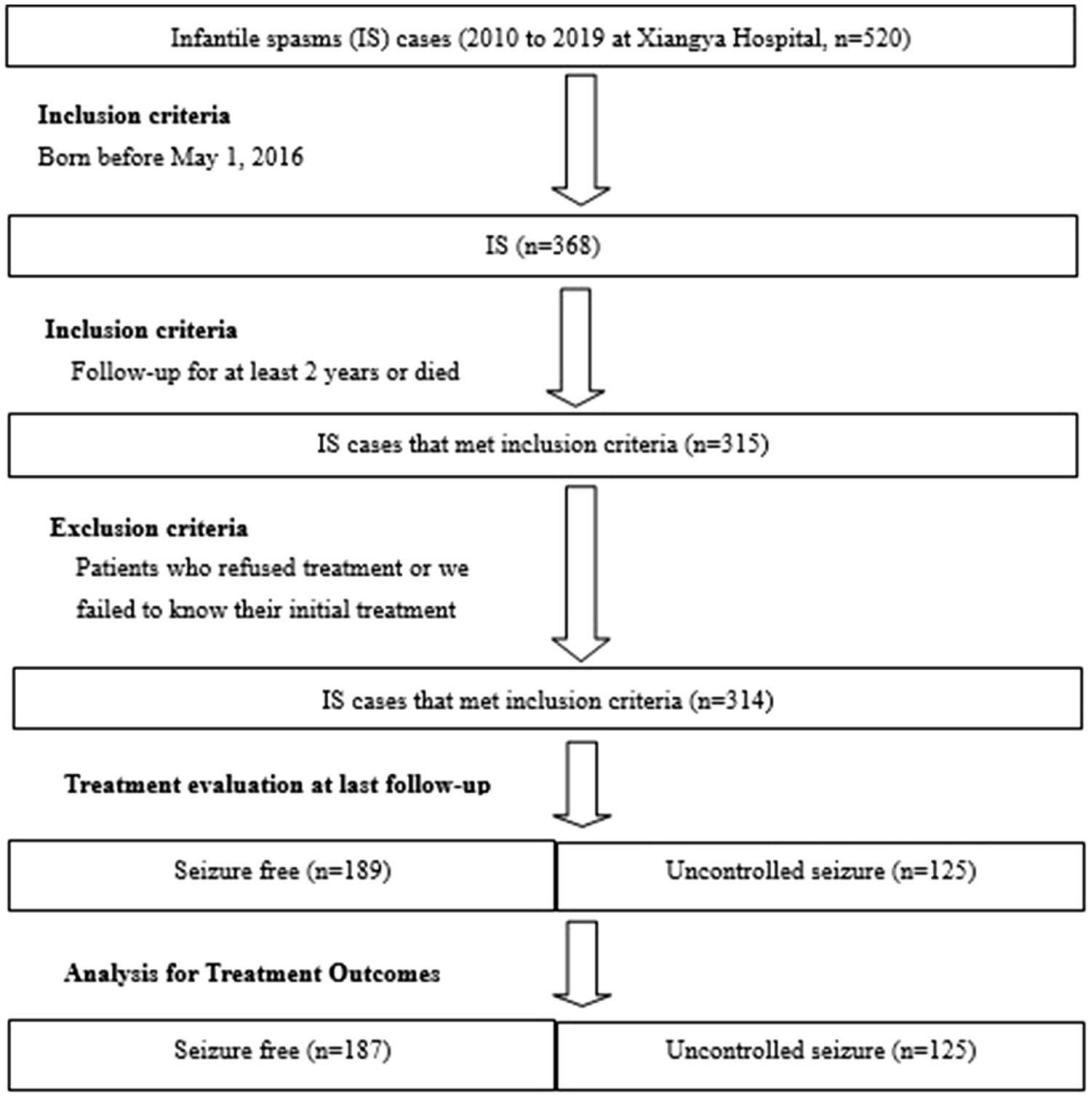
Flow chart for selection of cases with infantile spasms (ISs).

A total of 520 ISs cases were registered between 2010 and 2019. Of those 520 cases, 314 met the inclusion criteria (Fig. 1). Among those 314 cases, 64% (201) were males, and 60.19% (189) achieved seizure freedom. The medians (and respective interquartile ranges) of the time for seizure onset, from symptoms onset to treatment initiation, from symptoms onset to first-line treatment initiation, and of the age at last follow were 5.5 months (range, 3.5–8), 1 month (range, 0–2.5), 3 months (range, 1–6.45), and 68 months (range, 45–89), respectively. Later onset of spasms (median, 5.5 months), early treatment initiation (median, 1 month), and early first-line therapy initiation (median, 3 months) showed statistically significant association with seizure freedom (Table 1).

**Table 1:** Clinical characteristics of patients with infantile spasms stratified by 1-year seizure freedom.

Characteristics	No. (%)		P value
	Seizure free (n = 189)	Uncontrolled (n = 125)	
**Sex**			0.808
Male	122 (64.4)	79 (63.2)	
Female	67 (35.6)	46 (36.8)	
**Age at last follow-up, m**			0.086
Median (IQR)	68 (45–89)	59 (42–85)	
Range	25–129	5–172	
**Age at onset, m**			0.023
Median (IQR)	5.5 (3.5–8)	4 (2–7.5)	
Range	0–23.6	0–23	
**Median time between onset and treatment initiation, m**			0.05
Median (IQR)	1 (0–2.5)	1.9 (0–3.75)	
Range	0–19	0–34	
**Median time between onset and first-line therapy initiation, m**			0.003
Median (IQR)	3 (1–6.45)	4.5 (2–15)	
Range	0–89.5	0–76	
**Etiology**			0.445
Genetic	19 (10.1)	20 (16)	
Structural-congenital	8 (4.2)	4 (3.2)	
Structural-acquired	46 (24.3)	36 (28.8)	
Structural-genetic	9 (4.8)	4 (3.3)	
Metabolic	5 (2.6)	1 (0.8)	
Infectious	1 (0.5)	0	
Unknown	101 (53.4)	60 (48)	

%: percentage; IQR: interquartile range; m: months; No: numbers.

### Treatment outcomes

Although 314 cases met inclusion criteria, only 312 adhered to the therapies, and a total of 11 regimens were prescribed to them. At the last follow up, 59.94% (187/312) of the cases had been seizures free for ≥ 12 months. Of those 187 cases, 182 had both clinical and electrographic seizure freedom while 5 continued to have hypsarrhythmia in EEGs. Of those 187 cases, 169 (90.37%) used combination therapies and the remainder received monotherapies. Furthermore, the seizure freedom of 126 (67.4%) cases resulted from first-line therapies whereas, the remained cases’ seizure freedom resulted from other treatment strategies. Of those 312 cases, 9% (28) stopped AEDs because of side effects. In addition, 7% (12/171) of the cases who received ACTH therapy stopped it due to side effects. However, of those 12 cases, 11 (91.7%) continued with either oral prednisone or received the second course of ACTH.

Tuberous sclerosis complex (TSC) was absent for 95.5% (298/312) of the cases. Of those 298 cases, 146 (49%) received hormonal therapy as first or second regimen. Nevertheless, AEDs were added as combination therapies when there was a sign of a failure. As a result, 93 of those 146 (63.01%) cases had been seizure free for ≥ 1 year. The efficacy of each regimen in achieving seizure freedom was: first; 15.07%, second; 21.92%, third; 10.27%, fourth; 6.16%, fifth; 5.48%, sixth; 1.37%, seventh; 2.05%, eighth; 0.68%, ninth; 0.00%, tenth; 0.00%, and eleventh; 0.68%. The second course of ACTH or VGB was introduced in the third or fourth or fifth regimens for 32 cases. Consequently, of those 32 cases, 14 (44%) achieved seizures control. Overall, the efficacy of top five regimens in controlling seizures was 92.47% (86/93). Remarkable, the efficacy of each regimen diminished with the number of regimens received after the second regimen (Table 2a).

**Table 2:** Rates of 1-year seizure freedom with successive antiepileptic treatment regimens.

Successive antiepileptic regimens	Total patients trying these regimens, no	Seizure freedom			
		Total, no	% of patients achieving seizure freedom with treatment regimen	% of the total achieving seizure freedom (n = 93)	% of the total study cohort (n = 146)
**A: Non-tuberous sclerosis complex patients who received hormonal therapy as first or second regimen**					
First	146	22	15.07	23.66	15.07
Second	124	32	25.81	34.41	21.92
Third	88	15	17.05	16.13	10.27
Fourth	68	9	13.24	9.68	6.16
Fifth	49	8	16.33	8.60	5.48
Sixth	26	2	7.69	2.15	1.37
Seventh	16	3	18.75	3.23	2.05
Eighth	8	1	12.50	1.08	0.68
Ninth	4	0	0.00	0.00	0.00
Tenth	3	0	0.00	0.00	0.00
Eleventh	2	1	50.00	1.08	0.68
Total	146	93	N/A	100.00	63.01

Successive antiepileptic regimens	Total patients trying these regimens, no	Seizure freedom			
		Total, no	% of patients achieving seizure freedom with treatment regimen	% of the total achieving seizure freedom (n = 111)	% of the total study cohort (n = 170)
**B: Patients who received one of the first-line therapies as the first or second regimen**					
First	170	30	17.65	27.03	17.65
Second	140	36	25.71	32.43	21.18
Third	100	20	20.00	18.02	11.76
Fourth	75	10	13.33	9.01	5.88
Fifth	54	8	14.81	7.21	4.71
Sixth	29	2	6.90	1.80	1.18
Seventh	17	3	17.65	2.70	1.76
Eighth	9	1	11.11	0.90	0.59
Ninth	5	0	0.00	0.00	0.00
Tenth	3	0	0.00	0.00	0.00
Eleventh	2	1	50.00	0.90	0.59
Total	170	111	N/A	100.00	65.29

Successive antiepileptic regimens	Total patients trying these regimens, no	Seizure freedom			
		Total, no	% of patients achieving seizure freedom with treatment regimen	% of the total achieving seizure freedom (n = 76)	% of the total study cohort (n = 142)
**C: Patients who did not receive one of the first-line therapies as the first or second regimen**					
First	142	9	6.34	11.84	6.34
Second	131	9	6.87	11.84	6.34
Third	110	24	21.82	31.58	16.90
Fourth	81	12	14.81	15.79	8.45
Fifth	58	10	17.24	13.16	7.04
Sixth	40	3	7.50	3.95	2.11
Seventh	27	4	14.81	5.26	2.82
Eighth	16	4	25.00	5.26	2.82
Ninth	7	1	14.29	1.32	0.70
Tenth	3	0	0.00	0.00	0.00
Eleventh	1	0	0.00	0.00	0.00
Total	142	76	N/A	100.00	53.52

%: percentage; No: numbers; n: sample size.

One hundred and eleven cases out of the 170 (65.29%) who received one of the first-line therapies as first or second regimen achieved 1 year seizure freedom. The trend of response rates was similar as for those who used hormonal therapy as first or second regimen (Table 2b). There was a significant difference in seizure freedom rate between cases that received one of the first-line therapies as first or second regimen and those who received other therapies prior to first-line options (*P* = 0.035). Cases who did not receive one of the first-line therapies as first or second regimen accounted for 45.5% (142/312) of all cases (Table 2c). Consequently, the trend of response rate differed from the aforementioned two groups (Table 2a,b). The high proportions of seizure control were noticed in the regimens where first-line therapies were administered; 16.9% (18/24), 8.45% (8/12) and 7.04% (6/10) for third, fourth and fifth regimens, respectively. The overall outcome of all regimens was 53.52% (76/142) (Table 2c).

We also investigated the rate of 1 month seizure freedom for non-TSC cases who received hormonal therapy as first or second regimen. In one side, 78 (53.42%) of the 146 cases who received one course of ACTH achieved 1-month seizure freedom, and on the other side, 11 (32.35%) of the 34 cases who received two courses of ACTH achieved 1-month seizure freedom, notably, the difference between these two groups was statistically significant (*P* = 0.027). Likewise, there was a statistically significant difference between cases that received two courses of ACTH and those who used other therapies following the failure of the first course (*P* = 0.026).

### Response to successive treatment

Cases that did not achieve 1 year of seizure freedom by taking first regimen had a likelihood of uncontrolled seizures for each additional therapy tried (odds ratio [OR], 1.76; 95% CI 1.51–2.04), after adjusting for etiology, sex, age at onset, and the duration of seizures before treatment. The likelihood of becoming seizure-free diminished with each unsuccessful regimen according to survival analysis overtime.

**Figure 2:**
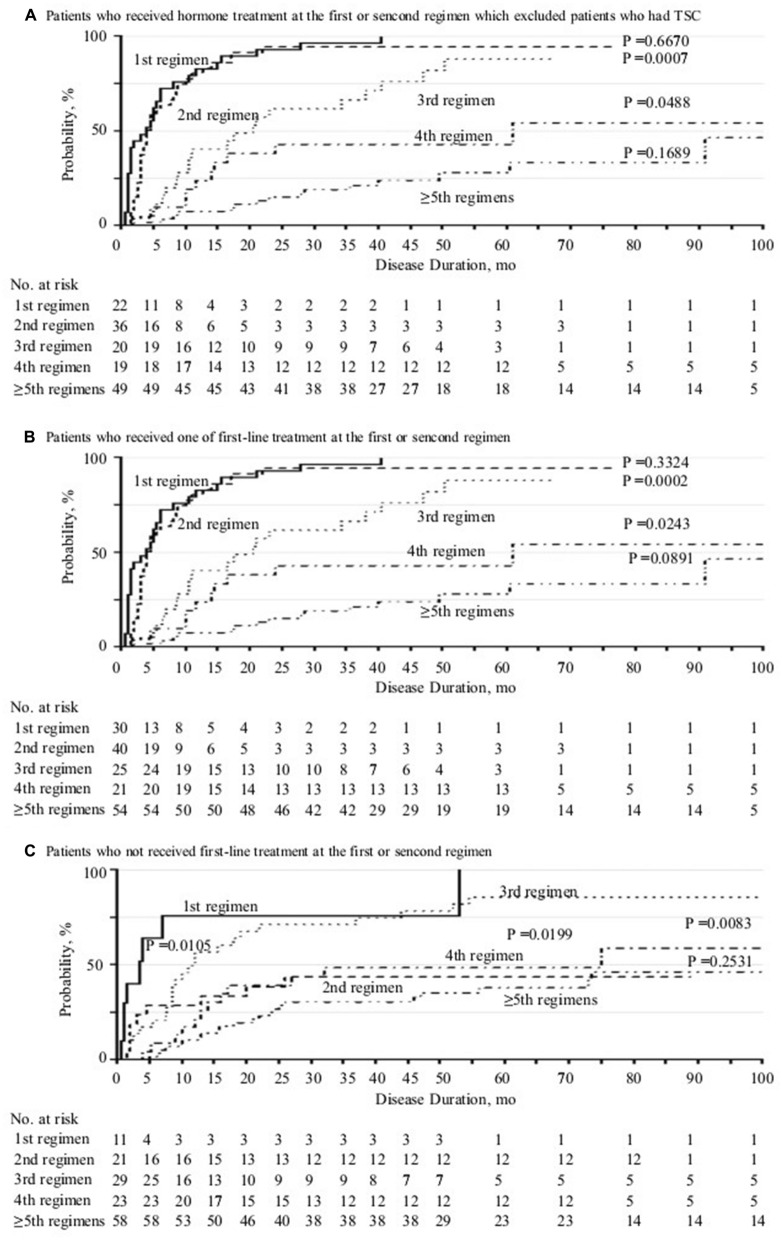
Cumulative probability of 1-year seizure freedom by disease duration and number of antiepileptic regimens tried.

There was a statistically significant difference in probability of seizure freedom between patients treated with the second and third regimens (HR, 0.34; 95% CI 0.20–0.60, *P* = 0.0007), and the third and fourth regimens (HR, 0.47; 95% CI 0.21–0.98, *P* = 0.0488) for non-TSC patients who received hormonal therapy as first or second regimen (Fig. 2A). There was a similar tendency in different regimens for the cases who used one of the first-line therapies as first or second regimen (Fig. 2B). There was a statistically significant difference in probability of seizure freedom between patients treated with the first and second regimens (HR, 0.22; 95% CI 0.07–0.70, *P* = 0.0105) as well as the third and fourth regimens (HR, 0.41; 95% CI 0.21–0.79, *P* value = 0.0083) for the patients who did not receive one of the first-line therapies as first or second regimen (Fig. 2C).

**Figure 3:**
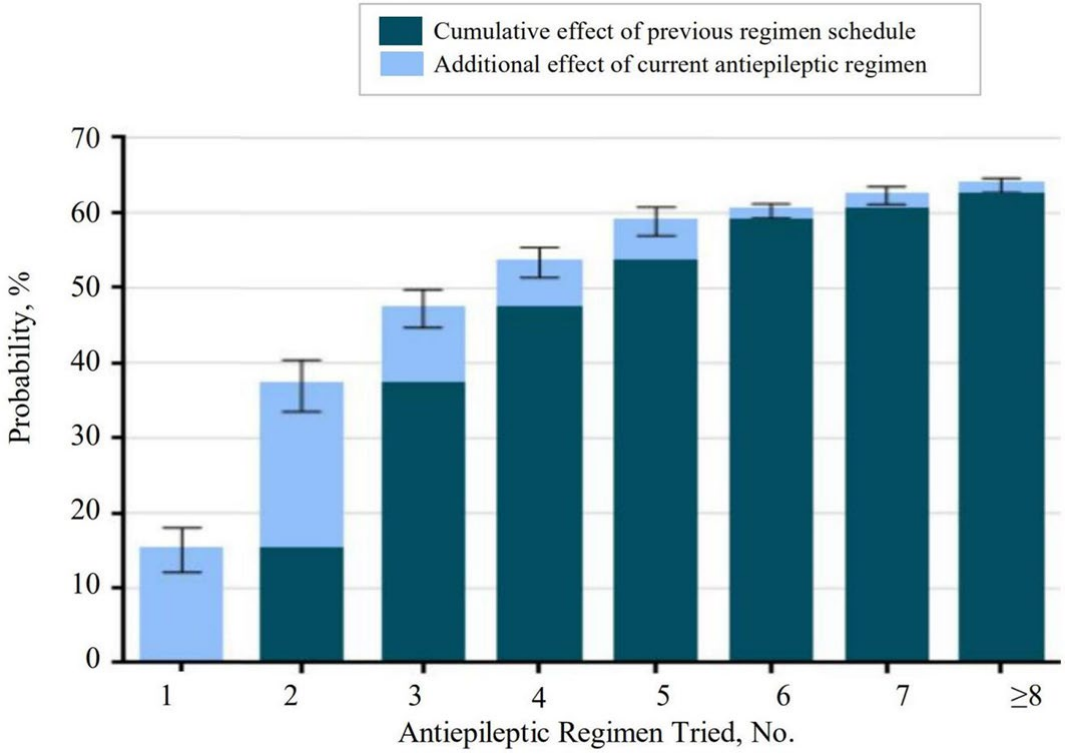
Increases in Probability of 1-Year Seizure Freedom for Each Additional Antiepileptic Regimen Tried in non-tuberous sclerosis complex patients who received hormonal therapy as first or second regimen. The percentage of patients achieved seizure freedom after receiving first, second, third, fourth, fifth, sixth, seventh and eighth regimens were 15.1%, 21.9%, 10.3%, 6.2%, 5.5%, 1.4%, 2.1% and 1.4%, respectively. Please see Table 2a for numbers of patients who achieved seizure freedom for each subgroup.

The effect of additional seizure control for non-TSC patients who received hormonal therapy as the first or second regimen decreased with each successive regimen tried after the first two standard therapies (Fig. 3). Twenty-two cases became seizure free for ≥ 1 year with the first regimen, so the predicted probability was 15.1% (95% CI 12.1–18.1%). If the first regimen failed, the second regimen could provide an additional 21.9% probability of seizure freedom (n = 32; 95% CI 18.5–25.3%), the third regimen could offer an additional 10.3% likelihood of seizure freedom (n = 15; 95% CI 7.8–12.8%), and the fourth regimen could give an additional 6.2% probability of seizure freedom (n = 9; 95% CI 4.2–8.2%). The fifth regimen could provide only a 5.5% additional likelihood of seizure freedom if the first four regimens failed to control all seizures. Each additional regimen from the sixth onwards, only added ≤ 2% probability of seizure freedom. The cumulative probabilities of seizure freedom were not significantly different with each successive regimen after trial of three regimens.

### Determinants of good psychomotor development

Thirty-seven of the 314 cases (11.78%) had good psychomotor development (normal or mild developmental delay) while 88.2% (277/314) had poor psychomotor development (moderate/severe developmental delay or deaths). Good prognosis was statistically significantly associated with; number of treatment regimens (2 (range, 1–9), *P* =  < 0.001), utilisation of first-line therapies as first or second regimen (27 (73%), *P* = 0.019), seizure freedom (37 (100%), *P* =  < 0.001), long duration of seizure control (3.58 years (range, 1.5–10), *P* =  < 0.001), and seizures controlled by the first-line therapies (30 (81.1%), *P* = 0.019) (Table 3).

**Table 3:** Factors affecting psycho-motor development.

Variable	Psycho-motor prognosis		P value	OR (95% CI)
	Poor prognosis (n = 277)	Good prognosis (n = 37)		
Sex (male:female)	178:99	23:14	0.805	–
Age at onset, m	3 (0–23.6)	5.5 (0.7–14)	0.632	–
Number of treatment regimens (Med (Min–Max))	4 (1–11)	2 (1–9)	< 0.001	0.599 (0.470–0.764)
Median time between onset and treatment initiation, m (Med (Min–Max))	1 (0–34)	1 (0–10)	0.602	–
Patients who received one of the first-line therapies as first or second regimen (%)	144 (52.0)	27 (73.0)	0.019	2.494 (1.163–5.348)
Patients who received first-line therapies (%)	234 (84.5)	35 (94.6)	0.099	–
Median time between onset of symptoms and first-line therapy initiation, median (Min–Max)	3.5 (0–89.5)	2 (0–27)	0.116	–
Seizure freedom (%)	152 (54.9)	37 (100)	< 0.001	–
Maximum control time, y (Med (Min–Max))	2.67 (1–7.83)	3.58 (1.5–10)	< 0.001	1.482 (1.219–1.803)
Age at seizure control, y (Med (Min–Max))	1.33 (0.33–8.92)	0.75 (0.33–6.75)	0.068	–
Seizures controlled by first-line therapies (%)	91 (59.9)	30 (81.1)	0.019	2.873 (1.186–6.956)

%: percentage; n: sample size; OR: odds ratio; CI: confidence interval; m: months; Med: median, Min = minimum; Max: maximum; y: years.

## Discussion

Regardless of many treatment options for ISs, the overall treatment outcome is still poor as only 59.94% achieved 1 year seizure freedom even after receiving 11 different regimens. Most patients achieved seizure control by combination therapy. The utilisation of one of the first-line therapies as first or second regimen is of beneficial.

Sixty-three percent of non-TSC patients who received hormonal therapy as first or second regimen achieved 1-year seizure freedom. Majority of the cases became seizure free with the first five regimens. The 6–11th regimens added 4.78% increment of seizure freedom (Table 2a). Similar trend was observed for the cases who received one of the first-line therapies as first or second regimen (Table 2b), however, it was different for the cases who did not do so. The trend of response rate was variable with no obvious pattern. Nevertheless, the high proportions of seizure control were noticed in the regimens where standard therapies were given including the third, fourth and fifth regimens (Table 2c). In addition, there was a statistically significant difference in seizure freedom rate (*P* = 0.035) between cases who received one of the first-line therapies as first or second regimen and those who did not do so. Our hospital is the largest epileptic referral center in Central South China thus, some of our cases were transferred from other hospitals. As a result, many of them did not receive first-line therapies as the first or second regimen because they were not available in those hospitals. Of the cases who achieved ≥ 1 year seizure freedom, 67.41% received first-line therapies whereas, 32.6% used other treatment options. Knupp et al. investigated the treatment outcome of ISs patients after 3 months of follow-up^14^. Their study revealed that 46% of the cases who received one of the first-line therapies showed good response and only 9% of the cases who received other therapies showed good response^14^. Altogether, our study plus previous studies show that the first-line therapies are superior to non-first-line therapies. Non-first-line therapies decrease the chances of seizure freedom. To our knowledge, this is the first study that tried to determine the trends of response rates for many regimens at once.

Other studies investigated the efficacy of one or two regimens at once, and the outcomes were assessed after a short-term follow-up^5,12,15^. Fifty-seven percent of the cases who received ACTH in the International Collaborative Infantile Spasms Study achieved seizure freedom^16^. Noteworthy, cases were assessed after a short period of follow-up^16^. Similarly, 53.42% of our cases achieved 1-month seizure freedom after receiving ACTH as first or second regimen. Moreover, there was a statistically significant difference between cases that received two courses of ACTH and those who received one course. Besides, there was a statistically significant difference between cases that received second course of ACTH and those who received other treatment options in the third regimen, following the failure of the first course of ACTH. Oguni et al. reported that 25% (2/8) of their cases achieved seizure remission after receiving the second course of ACTH^17^. Likewise, second courses of ACTH were given following the failure of several AEDs, as a result, 75% of cases who presented with focal ISs, and 83% of the cases who presented with diffuse ISs achieved remission (5/6)^18^. Although the second course of ACTH seems to be useful, time for commencement is not clear.

In this study, the high rate of seizure freedom was noticed among cases who received combination therapies (90.37%) as compared to those who received monotherapies (9.63%). Zou, et al. reported in their randomized controlled trial that ACTH + magnesium sulfate is superior to ACTH alone^19^. Similar findings were reported by the International Collaborative Infantile Spasms Study; the combination of ACTH and VGB is superior to ACTH alone^16^. Altogether, previous studies plus our study emphasize the importance of combination therapies. Nevertheless, it is important to note that, of the 187 cases who achieved seizure freedom, 182 had both clinical and electrographic seizure freedom while 5 continued to have hypsarrhythmia in EEGs. Likewise, other studies have reported high rates of persistence of hypsarrhythmia on video EEG in cases where no clinical spasms are reported by the caretaker^16–19^.

Our study has revealed that later onset of spasms, early treatment initiation and early first-line therapies initiation are determinants of seizure freedom which correlate with findings from other studies^20–22^. Our cohort consisted of children aged above 3 years thus, we were able to assess their psychomotor development. We selected this particular group of patients because their developmental milestones could be evaluated by the questionnaire at the last follow-up. The scale we used could exclude age confounders in children's developmental assessment than the Modified Rankin Scale (MRS) score. Although 60.19% (189) of our cases achieved seizure freedom, only 11.78% (37) were seizure-free and had good psychomotor development. Similarly, Iype et al. reported that although 51.2% of their cases were seizure free, only 11.3% were seizure-free and had normal development^23^. Fewer numbers of treatment regimens, utilisation of first-line therapies as first or second regimen, seizure freedom, long duration of seizures control and seizures controlled by first-line therapies were associated with good psychomotor development. Iype et al. attempted to find determinants of good psychomotor development but they found none^23^. Thus, early utilisation of first-line therapies can increase the chances of longer term developmental outcomes. In contrast, early utilisation of non-first-line therapies decreases the chances for both seizure freedom and for longer term developmental outcomes.

### Limitations

Despite the fact that our study is the first one to assess long-term outcomes of many regimens (n = 11) at once, it has some limitations that deserve to be mentioned. Although there are established treatment for ISs based on higher efficacy than others only 146 cases received first-line therapies first. In addition, this study was retrospective in nature thus, hamper the ability to effectively assess drug efficacy. Future studies may asses the patterns of response rates of many regimens prospectively. Moreover, first-line therapies should be administered first whenever possible.

## Conclusions

Most patients achieve seizure control by combination therapies. The long-term seizure freedom diminishes with each unsuccessful regimen (> 2 regimens) for the cases receiving first-line therapy first. The seizure freedom is less likely to be observed after the fifth regimen. Seizure freedom rate is higher for the cases who receive first-line therapy on time. Only few cases among those who achieve seizure freedom remain with good psychomotor development. The utilisation of first-line therapies first, seizure freedom, long duration of seizures control, and seizures controlled by first-line therapies seem to associate with good prognosis.

## Methods

### Participants

Infants diagnosed with ISs at epileptic center of Xiangya Hospital, Central South University were retrospectively evaluated for eligibility for this study. The inclusion criteria were patients characterized by (1) an ascertained diagnosis of ISs confirmed by epileptic spasms and an initial EEG demonstrating features of hypsarrhythmia or atypical hypsarrhythmia with or without developmental regression according to the new 2017 International League Against Epilepsy classification^24^, (2) born before May 1, 2016, (3) record of follow-up for at least 2 years or death. We excluded cases without information related to initial treatment and/or refused drugs.

Data were analysed and reported anonymously based on patient medical files using a structured questionnaire developed for the purposes of this study. Clinical information was obtained from medical records or the questionnaire or from parents or refereeing institutions. The following information were collected for analysis: sex, date of birth, age of spasms onset, time from spasms onset to start of treatment, and time from spasms onset to initiation of first-line treatment (hormonal treatment and/or VGB).

### Treatments

Treatment was initiated after two or more seizures^25^. The selection of an appropriate AEDs was done according to seizure type, drug interactions as well as their side effects^26^. Furthermore, cost, adverse effects, availability of recommended drugs and parental choice were considered in selecting an appropriate therapy for options such as hormonal treatment, VGB, ketogenic diet, traditional Chinese medicine, vagus nerve stimulation (VNS) and surgery. The first-generation AEDs such as phenytoin, phenobarbitone, carbamazepine, oxcarbazepine and VPA, and the second generation AEDs such as LEV, TPM, lamotrigine, nitrazepam, clonazepam, clobazam, zonisamide and rufinamide were prescribed. ACTH, intravenous pulse methylprednisolone, and prednisone were the main hormonal therapies provided. Every AED given was titrated slowly from low to high dosage, and the appropriate dosage was maintained for at least 1 month. The amount of drugs used and time exposures to treatments for our patients were as follows; TPM 2–5 mg/kg/day for ≥ 2 months, LEV 28–60 mg/kg/day for ≥ 3 months, VPA 20–44 mg/kg/day for ≥ 5 months, and VGB 100–112 mg/kg/day for ≥ 1 month. ACTH injection 1–4 U/kg/day was administered for more than 14 days^27^. Therapies prescribed for short duration were excluded from the list of regimens regardless of whether they were tolerable or not. We excluded the AEDs, VGB, prednisone and VNS used for less than 1 month, ACTH injection used for the minimum of 14 days, and ketogenic diet used for less than 3 months. A combination therapy was used as an initial or later choice despite previous administration of one or more monotherapies^28^.

### Follow-up and ascertainment of outcomes

The frequency of follow-up was at the discretion of the attending neurologist, therefore, it varies among cases. Client's response to therapy as well as other clinical information were recorded at each follow-up visit. Patients’ parents and guardians were asked to record the frequency of spasms or other types of seizures that occurred between the visits. Moreover, caretakers and primary care physicians could contact us via a dedicated telephone line or videoconferencing system. Noteworthy, all cases achieved clinical seizure freedom received 1–2 EEG examinations per year to add-on clinical assessment. All patients were followed up prospectively until March 2019, or until their deaths.

### Definitions

Causes of ISs were classified according to the National Infantile Spasms Consortium guideline^29^. They were broadly classified as genetic, genetic-structural, structural-congenital, structural-acquired, metabolic, immune, infectious, and unknown.

Seizure freedom was defined as a patient experiencing no seizures for the previous 12 months or longer after receiving an intervention^30^. Every patients would have EEG at least 1–2 times every year. Oftentimes, the EEG recording was done for 15 h; it started from 4:30 p.m. to 7:30 a.m. in the next day. Besides, four hours EEG recording was done rarely. However, we made sure that the EEG recording captured sleep in both situations. The combination of clinical assessment plus video EEG recording was used to monitor seizure freedom. Time to seizure freedom was expressed as the time from treatment initiation to the day that patient became seizure-free (i.e., the first day after seizures-free). Treatment responses for the expired or lost to follow-up cases were assessed as of the last clinical visit.

A regimen was defined as a single therapy (a single drug or surgery or others). Hormonal therapy or VGB was prescribed as a first-line option in most of the patients, however, it was not always used as first regimen. Oftentimes, ACTH or prednisone or TPM or LEV or VPA alone or in combination were chosen as first or second regimen. Whereas, third and above regimens were adjusted according to the etiology^31,32^, notable, second course of ACTH, or VGB or ketogenic diet were often tried.

Other seizure types that raised concurrently or sequentially with spasms were evaluated before the onset of symptoms. In addition, developmental milestones were assessed according to the National Infantile Spasms Consortium guideline^29^, and recorded based on provider’s perception of overall development, motor, and cognitive status. Developmental milestones were categorized as normal, equivocal, mild, or definite abnormality. A patient without documented abnormalities was considered to have normal development. The term “mild developmental delay” was used when one domain was abnormal, “moderate delay” was used if two or more domains were marked as mild or one domain was marked as a definite abnormal. Lastly, the term “severe delay” was used when two or more domains were marked as definite abnormal.

### Statistical analysis

The data was processed by SPSS Version 22 software. The Pearson χ^2^ test and the Mann–Whitney test were used to compare categorical and non-parametric continuous data, respectively. The logistic regression was carried out to assess the association between the number of treatment regimens prescribed and the outcomes. Survival analysis was done in order to determine the difference in the time required for the use of each regimen to result in seizure freedom. Cox regression was carried out to estimate the time for the patients to become seizure free while taking each regimen.

### Ethical standards

The informed and written consent requirements were waived by the Institutional Ethics Committee of Xiangya Hospital, Central South University because all data were deidentified prior to analysis. Moreover, data were anonymously analysed and reported. This study was reviewed and approved by the Institutional Ethics Committee of Xiangya Hospital Central South University, thus complying with the treaty agreed to in 1964 in Helsinki by the World Medical Association on ethical principles of human research for medical purposes and subsequent revisions of the same (2013).

## Data Availability

The data that support the findings of this study are available on request from the corresponding author.
